# The Significance of Endothelial Dysfunction in Long COVID-19 for the Possible Future Pandemic of Chronic Kidney Disease and Cardiovascular Disease

**DOI:** 10.3390/biom14080965

**Published:** 2024-08-08

**Authors:** Hidekatsu Yanai, Hiroki Adachi, Mariko Hakoshima, Hisayuki Katsuyama, Akahito Sako

**Affiliations:** 1 Department of Diabetes, Endocrinology and Metabolism, National Center for Global Health and Medicine Kohnodai Hospital, 1-7-1 Kohnodai, Ichikawa 272-8516, Chiba, Japan; dadachidm@hospk.ncgm.go.jp (H.A.); d-hakoshima@hospk.ncgm.go.jp (M.H.); d-katsuyama@hospk.ncgm.go.jp (H.K.); 2 Department of General Medicine, National Center for Global Health and Medicine Kohnodai Hospital, 1-7-1 Kohnodai, Ichikawa 272-8516, Chiba, Japan; dsako@hospk.ncgm.go.jp

**Keywords:** atherosclerotic cardiovascular disease, chronic kidney disease, endothelial dysfunction, long coronavirus disease 19

## Abstract

Various symptoms have been reported to persist beyond the acute phase of severe acute respiratory syndrome coronavirus 2 (SARS-CoV-2) infection, which is referred to as long coronavirus disease 19 (long COVID-19). Over 65 million individuals suffer from long COVID-19. However, the causes of long COVID-19 are largely unknown. Since long COVID-19 symptoms are observed throughout the body, vascular endothelial dysfunction is a strong candidate explaining the induction of long COVID-19. The angiotensin-converting enzyme 2 (ACE2), the entry receptor for SARS-CoV-2, is ubiquitously expressed in endothelial cells. We previously found that the risk factors for atherosclerotic cardiovascular disease (ASCVD) and a history of ASCVD raise the risk of severe COVID-19, suggesting a contribution of pre-existing endothelial dysfunction to severe COVID-19. Here, we show a significant association of endothelial dysfunction with the development of long COVID-19 and show that biomarkers for endothelial dysfunction in patients with long COVID-19 are also crucial players in the development of ASCVD. We consider the influence of long COVID-19 on the development of chronic kidney disease (CKD) and ASCVD. Future assessments of the outcomes of long COVID-19 in patients resulting from therapeutic interventions that improve endothelial function may imply the significance of endothelial dysfunction in the development of long COVID-19.

## 1. Introduction

A broad range of symptoms has been reported to persist beyond the acute phase of severe acute respiratory syndrome coronavirus 2 (SARS-CoV-2) infection, which is referred to as “long coronavirus disease 19 (long COVID-19)” [1,2,3]. Preliminary reports have shown that at least 65 million individuals are estimated to suffer from long COVID-19, with cases increasing daily [4,5]. SARS-CoV-2 invades human cells via angiotensin-converting enzyme 2 (ACE2) and injures multiple organs [2]. Typical symptoms of long COVID-19 include fatigue/malaise, joint pain, muscle pain, cough, sputum, shortness of breath, chest pain, hair loss, memory impairment, decreased concentration, headache, depression, olfactory dysfunction, taste disturbance, palpitations, diarrhea, abdominal pain, sleep disturbances, and muscle weakness [6,7]. The causes of long COVID-19 are largely unknown. Since the symptoms of long COVID-19 are observed throughout the body, it can be said that disorders in blood vessels distributed throughout the body undergoing short, vascular endothelial dysfunction may be a strong candidate for the cause of long COVID-19. ACE2, the entry receptor of SARS-CoV-2, is ubiquitously expressed in endothelial cells [8,9], suggesting the possible contribution of endothelial dysfunction to the development of long COVID-19. We previously reported that the risk factors for atherosclerotic cardiovascular disease (ASCVD), such as diabetes and hypertension and a history of ASCVD, are risk factors for severe COVID-19 [10], suggesting that pre-existing endothelial dysfunction, which involves an initial lesion of atherosclerosis, may be associated with the development of severe COVID-19. 

Here, we will prove the significant association of endothelial dysfunction with the development of long COVID-19 and show that the biomarkers for endothelial dysfunction in patients with long COVID-19 are crucial players in the development of ASCVD. We will consider the influence of long COVID-19 on atherosclerotic risk factors such as diabetes and hypertension, and on the development of chronic kidney disease (CKD) and ASCVD. Furthermore, we will suggest therapeutic interventions for long COVID-19, considering vascular endothelial dysfunction as a treatment target for long COVID-19.

## 2. The Association of Pre-Existing Endothelial Dysfunction with the Development of Severe COVID-19

Diabetes and hypertension are important risk factors for ASCVD and induce endothelial dysfunction, which is an early lesion in atherosclerosis. To understand the association of ASCVD risk factors inducing endothelial dysfunction with the development of severe COVID-19, we previously performed a meta-analysis by using PubMed. In this meta-analysis, the prevalence of diabetes in severe patients was found to be significantly higher than that in non-severe patients (odds ratio (OR), 3.52; 95% confidence interval (CI), 2.65 to 4.67), and the prevalence of hypertension in severe patients was significantly higher than that in non-severe patients (OR, 2.69; 95% CI, 2.16 to 3.34) [10]. The prevalence of ASCVD in severe COVID-19 patients was significantly higher than that in non-severe patients (OR, 5.37; 95% CI, 3.73 to 7.74), suggesting a significant association between vascular damage and severity in COVID-19.

Obesity is also an important ASCVD risk factor. Patients with overweight and obesity admitted into a medical ward for COVID-19, despite their younger age, required more frequently assisted ventilation and access to intensive care units (ICUs) than normal-weight patients [11]. ACE2 is the cellular entry receptor of SARS-CoV-2 [2], and increased ACE2 expression in the bronchial epithelium of overweight patients compared to non-overweight patients has been observed [12], indicating that SARS-CoV-2 is more likely to enter the human body in obese people as compared with non-obese people. It has been proposed that an increase in the secretion of interleukin-6 (IL-6) and tumor necrosis factor-alpha (TNF-α) in adipose tissue under conditions of obesity-induced insulin resistance could underlie the associations of insulin resistance with endothelial dysfunction and coagulopathy [13]. 

There is a close causal relationship between the chronic increase in circulating insulin levels and the onset of endothelial dysfunction [14]. Insulin resistance, as observed in metabolic syndrome, type 2 diabetes, arterial hypertension, and obesity, determines important alterations in circulatory homeostasis, such as via the synthesis of endothelin-1 (ET-1) over that of nitric oxide (NO), stimulating the sympathetic system and acting as a growth factor for the cells of the vascular wall [15], i.e., activating a whole series of factors that produce and worsen atherosclerosis. Increases in endothelial dysfunction, produced by acute COVID-19 and long COVID-19, unfortunately find fertile ground in these cases, resulting in a worsening of the evolution of the disease.

Insulin resistance is associated with the increased expression and secretion of plasminogen activator inhibitor-1 (PAI-1) by endothelial cells [13]. Von Willebrand factor (VWF) is elevated in insulin-resistant states, suggesting that insulin resistance induces endothelial dysfunction [13]. The elevation in inflammatory cytokines, endothelial dysfunction, and a procoagulant state are already present in obese people even before SARS-CoV-2 infection [16]. SARS-CoV-2 infection may enhance the elevation of inflammatory cytokines, which leads to a cytokine storm and induces further endothelial injury. Elevated PAI-1 and endothelial injury induce thrombosis. Multivariable regression remarkably increased the odds of in-hospital death associated with d-dimer (the marker for thrombosis) by greater than 1 µg/mL (18.42, 2.64–128.55; *p* = 0.0033) on admission [17]. An elevated VWF (the marker for endothelial injury) level was also observed in COVID-19 patients in the ICU [18]. Fibrinogen and d-dimer levels also increased in patients in the ICU; such patients showed hypercoagulability together with a severe inflammatory state [18] called “systemic severe coagulopathic vasculitis (SSCV)”, which is the most crucial factor causing severe COVID-19 [16]. 

**Figure 1:**
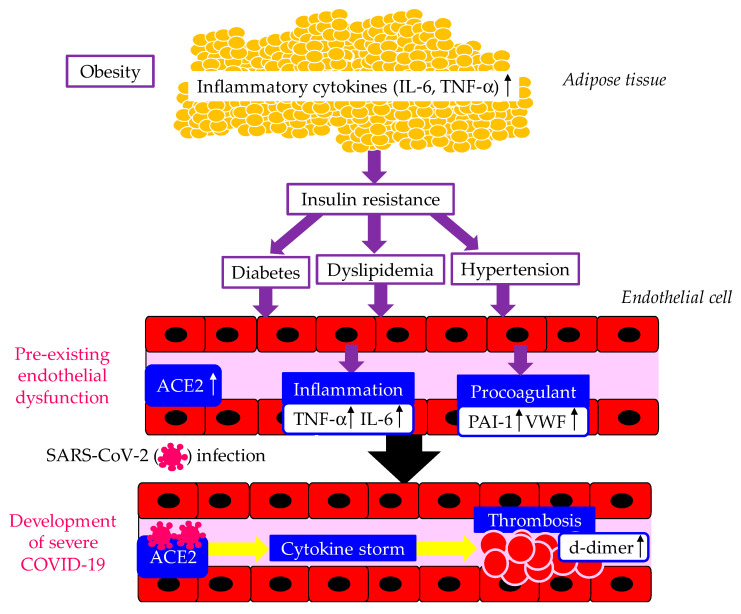
The mechanisms underlying the development of severe COVID-19 in patients with metabolic syndrome. Up arrows indicate increases in the expression of molecules, such as ACE2, angiotensin-converting enzyme 2; IL-6, interleukin-6; PAI-1, plasminogen activator inhibitor-1; TNF-α, tumor necrosis factor-alpha; VWF, von Willebrand factor.

The mechanisms underlying the development of severe COVID-19 in patients with metabolic syndrome are shown in Figure 1. Metabolic syndrome and its components, such as obesity, diabetes, and hypertension, are significantly associated with a susceptibility to SARS-CoV-2 infection and the severity of COVID-19 [19]. Enhanced ACE2 expression, pre-existing endothelial dysfunction, and a procoagulant state induced by adipocytokine dysregulation in metabolic syndrome may play crucial roles in the development of severe COVID-19 [19]. The secretion of inflammatory cytokines such as IL-6 and TNF-α is increased in patients with obesity. Increases in inflammatory cytokines induce insulin resistance, diabetes, dyslipidemia, and hypertension, which cause endothelial dysfunction. Dysfunctional endothelial cells release more IL-6, TNF-α, and PAI-1 and VWF, which induce highly inflammatory and procoagulant states in patients with obesity. The entry of SARS-CoV-2 via overexpressed ACE2 induces a cytokine storm and thrombosis, which lead to the development of severe COVID-19. A systematic review and meta-analysis showed that IL-6 and d-dimer levels were significantly elevated in patients with severe COVID-19 [20]. 

## 3. A Significant Association of Endothelial Dysfunction with the Development of Long COVID-19

The generation of abnormal levels of oxidants under a COVID-19-induced cytokine storm causes the irreversible oxidation of a wide range of macromolecules and subsequent damage to cells, tissues, and organs [21]. Clinical studies have shown that oxidative stress initiates endothelial damage, which increases the risk of complications in COVID-19 and post-COVID-19 or long COVID-19 cases [21].

Lasting effects and long-term sequelae could persist after the infection and may arise due to persistent endothelial dysfunction. The endothelial quality index in a large cohort of long COVID-19 patients was evaluated [22]. In a multivariate analysis, endothelial dysfunction was found to be one of the independent risk factors of long COVID-19 [22]. Long COVID-19 symptoms, specifically non-respiratory symptoms, occur due to persistent endothelial dysfunction. 

The endothelial function of patients hospitalized for COVID-19 was assessed by brachial-artery-flow-mediated dilation (FMD) [23]. FMD was significantly impaired in the COVID-19 group compared to that in the control. ICU-treated subjects presented a significantly impaired FMD compared to those treated in the medical ward. However, a significant improvement in FMD was noted during the follow-up; the FMD remained impaired compared to that in the control at 1 month and 6 months after hospital discharge.

The meta-analysis found that a total of 644 convalescent COVID-19 patients showed significantly lower FMD values as compared to 662 controls (MD [mean difference], −2.31%; 95% CI, −3.19 to −1.44; *p* < 0.0001) [24]. Similar results were obtained in the sensitivity analyses of studies involving participants in either group with no cardiovascular risk factors or history of coronary artery disease (CAD). Meta-regression models showed that an increasing prevalence of post-acute sequelae of COVID-19 was linked to a greater difference in FMD between cases and controls.

To determine whether endothelial cell activation may be sustained in convalescent COVID-19 patients and contribute to long COVID-19 pathogenesis, endothelial cell activation was assessed in COVID-19 patients [25]. Endogenous thrombin potential and thrombin were increased in convalescent COVID-19 patients. The biomarkers for endothelial dysfunction, including VWF, factor VIII, and soluble thrombomodulin levels, were significantly elevated in convalescent COVID-19 patients compared with those for the controls, suggesting that endothelial dysfunction may contribute to long COVID-19 pathogenesis. In another study, compared to those of the controls, long COVID-19 patients showed increased levels of the endothelial dysfunction indices L-selectin and P-selectin [26]. 

Several studies have found a persistence in vascular damage with increased circulating markers of endothelial dysfunction, coagulation abnormalities with heightened thrombin generation capacity, and abnormalities in platelet counts in long COVID-19 [27]. Platelets contribute to elevated levels of thrombo-inflammatory mediators and pro-coagulant extracellular vesicles in individuals with long COVID-19 [28].

## 4. The Biomarkers for Endothelial Dysfunction in Patients with Long COVID-19 as Risk Factors for ASCVD

**Figure 2:**
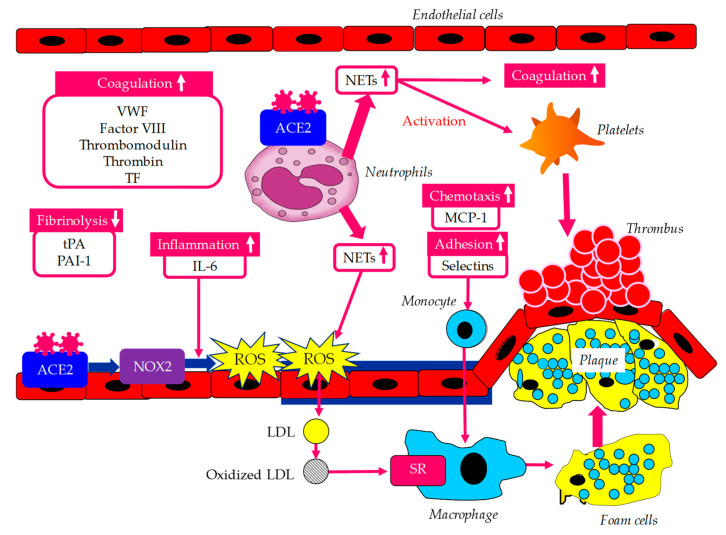
Various atherogenic factors induced by vascular endothelial dysfunction caused by infection with SARS-CoV-2. Up arrows indicate an increase in degree of atherogenic and thrombogenic factors. ACE2, angiotensin-converting enzyme 2; IL-6, interleukin-6; LDL, low-density lipoprotein; MCP-1, monocyte chemoattractant protein-1; NETs, neutrophil extracellular traps; NOX2, NADPH oxidase 2; PAI-1, plasminogen activator inhibitor-1; ROS, reactive oxygen species; SR, scavenger receptor; TF, tissue factor; tPA, tissue plasminogen activator; VWF, von Willebrand factor.

Various atherogenic factors induced by vascular endothelial dysfunction caused by infection with SARS-CoV-2 are shown in Figure 2.

### 4.1. NADPH Oxidase (NOX) 2 (NOX2), IL-6, and Monocyte Chemoattractant Protein-1 (MCP-1)

#### 4.1.1. The Association of NOX2, IL-6, and MCP-1 with Long COVID-19

The activation of NADPH-oxidase (NOX)-family oxidases induces endothelial dysfunction, while augmenting inflammation to further deteriorate endothelial cell barrier dysfunction/injury. Upon the binding of the spike protein (S protein) to its membrane receptor ACE2, the S protein stimulates NOX2-dependent reactive oxygen species (ROS) production [29]. Excessive ROS production by the S protein induces ROS-dependent cellular signaling, including the induction of cytokines such as IL-6 and MCP-1 [29]. IL-6 also induces ROS production in a NOX2-dependent manner, aggravating endothelial oxidative stress, which in turn sustains endothelial dysfunction. A meta-analysis showed that increased IL-6 correlates with long COVID-19, suggesting IL-6 as a basic determinant predicting long COVID-19 [30]. MCP-1 was found to be higher in long COVID-19 patients with the most frequent symptoms [31].

#### 4.1.2. The Association of NOX2, IL-6, and MCP-1 with ASCVD

The activation of vascular NOX and the production of ROS by these enzyme systems are common in ASCVD. Animal studies showed that the ROS produced by NOX2 contributes to ASCVD, including atherosclerosis and hypertension [32]. Recently, efforts have been directed at developing inhibitors of NOX that could provide useful experimental tools and might have therapeutic potential in the treatment of human diseases. Inflammatory processes are deeply implicated in the pathogenesis of ASCVD. A meta-analysis including 17 studies showed that baseline IL-6 levels were significantly higher in ASCVD cases than in non-ASCVD controls (MD, 0.36 pg/mL; 95%CI, 0.28 to 0.44 pg/mL) [33], suggesting that higher IL-6 levels in healthy individuals are associated with ASCVD risk. MCP-1 is mainly expressed by inflammatory cells and endothelial cells [34]. The expression level is upregulated after proinflammatory stimuli and tissue injury, which are associated with atherosclerotic lesions [34]. MCP-1 has been reported to play an important role in the pathogenesis of atherosclerosis. Genetic instruments for 41 cytokines and growth factors were obtained from a genome-wide association study (GWAS) of 8293 healthy adults [35]. A genetic predisposition to higher MCP-1 levels was associated with a higher risk of any stroke (OR per 1 SD increase, 1.06; 95% CI, 1.02–1.09; *p* = 0.0009), any ischemic stroke (OR, 1.06; 95% CI, 1.02–1.10; *p* = 0.002), large-artery stroke (OR, 1.19; 95% CI, 1.09–1.30; *p* = 0.0002), and cardioembolic stroke (OR, 1.14; 95% CI, 1.06–1.23; *p* = 0.0004). Genetically determined higher MCP-1 levels were further associated with CAD (OR, 1.04; 95% CI, 1.00–1.08; *p* = 0.04) and myocardial infarction (OR, 1.05; 95% CI, 1.01–1.09; *p* = 0.02).

### 4.2. VWF, Factor VIII, and ADAMST-13

#### 4.2.1. The Association of VWF, Factor VIII, and ADAMST-13 with Long COVID-19

The biomarkers for endothelial dysfunction, such as VWF, factor VIII, and soluble thrombomodulin levels, were significantly elevated in convalescent COVID-19 patients compared with those for the controls [25]. Plasma VWF facilitates platelet aggregation and adhesion to the sites of vascular injury, reinforcing the pro-coagulation effect of primary and secondary hemostasis. Vascular injury leads to the generation of a platelet plug at the site of injury, which is subsequently stabilized through the activation of the coagulation cascade and the formation of a cross-linked fibrin network. Platelet adhesion, activation, and aggregation, together with the concurrent thrombin generation, are central events in this response. VWF has been found to be elevated in COVID-19 patients, acting as a marker of acute and sustained endothelial cell activation and a predictor of poor outcomes [36,37]. The FVIII–VWF complex plays critical roles in regulating both platelet responses and the normal coagulation cascade [38]. VWF activity is largely regulated by ADAMST-13, which is generated in endothelial cells. ADAMST-13 cleaves VWF multimers, reducing their pro-adhesive and pro-coagulant activity [39]. A higher VWF/ADAMST-13 ratio, together with endothelial injury, coagulopathy, and poor prognosis, has been found in cases of acute and long COVID-19 [40,41,42,43]. 

#### 4.2.2. The Association of VWF, Factor VIII, and ADAMST-13 with ASCVD

VWF is involved in the pathogenesis of ASCVD. A meta-analysis using nine eligible studies including 576 cases and 632 controls showed that plasma VWF levels were significantly higher in type 2 diabetic patients with ASCVD than in type 2 diabetic patients without ASCVD (MD, 0.61; 95% CI, 0.32 to 0.90; *p* < 0.00001) [44]. Another meta-analysis showed that men in the top third of baseline VWF values (tertile cutoff > 126 IU/dL) had an OR for CAD of 1.83 (95% CI, 1.43 to 2.35; *p* < 0.0001) compared with those in the bottom third (tertile cutoff < 90 IU/dl), after adjustments for age [45]. 

Factor VIII and its carrier protein VWF are associated with the risk of arterial and venous thrombosis. Sabater-Lleal, M. et al. meta-analyzed genome-wide association results from 46,354 individuals of European, African, East Asian, and Hispanic ancestry [46]. Mendelian randomization suggested the causal effects of plasma FVIII activity levels on venous thrombosis and CAD risk, and of plasma VWF levels on ischemic stroke risk.

The meta-analysis based on published results confirmed a significant association of ADAMTS-13 levels with ischemic stroke (relative risk (RR), 2.72; 95% CI, 1.52 to 4.85, for low versus high ADAMTS-13 levels) [47]. The meta-analysis of individual patients’ data, performed in observational studies investigating the association between ADAMTS-13 levels and myocardial infarction, showed that low ADAMTS-13 levels were associated with myocardial infarction risk, with an OR of 1.89 (95% CI, 1.15 to 3.12) for values below the 5th percentile versus above, suggesting that low ADAMTS-13 levels are associated with an increased risk of myocardial infarction [48].

### 4.3. Tissue Plasminogen Activator (tPA) and PAI-1

#### 4.3.1. The Association of tPA and PAI-1 with Long COVID-19

Fibrinolytic activity is essential to dissolving the fibrin clot in tertiary hemostasis and is mainly determined by the balance between the endothelial tPA and PAI-1 [49]. Endothelial cells are centrally involved in maintaining blood fluidity and providing controlled vascular hemostasis at sites of injury [50]. Under physiological conditions, endothelial cells constitute a non-adhesive surface preventing the activation of platelets and a coagulation cascade [50]. Multiple fibrinolytic and antithrombotic properties act on their cell surface, contributing to the maintenance of blood fluidity [50]. SARS-CoV-2-infection-induced endothelial dysfunction may cause imbalances in fibrinolysis. Endothelial damage triggers the release of anti-fibrinolytic mediators such as PAI-1, which, in turn, inhibits fibrinolysis. In a study conducted by Yu Zuo et al., markedly elevated tPA and PAI-1 levels were observed in patients hospitalized with COVID-19. Both factors demonstrated strong correlations with neutrophil counts and markers of neutrophil activation. High levels of tPA and PAI-1 were associated with worse respiratory status [51]. Hypo-fibrinolysis, mainly associated with increased PAI-1 levels, was observed in COVID-19 patients admitted to an ICU [52]. COVID-19 patients showed significantly elevated levels of tPA and PAI-1 compared to the control group [53]. Higher levels of t-PA at the time of admission were associated with lower survival rates [53]. Persistent hypo-fibrinolysis has been reported to contribute to long COVID-19 manifestations [54].

#### 4.3.2. The Association of tPA and PAI-1 with ASCVD

Meta-analysis results have suggested that PAI-1 gene polymorphism is associated with an increased risk of ischemic stroke, myocardial infarction, and CAD [55,56,57]. In another meta-analysis, patients experiencing major adverse cardiovascular events (MACE) had higher PAI-1 antigen levels, with an MD of 6.11 ng/mL (95% CI, 3.27 to 8.96), suggesting that elevated plasma PAI-1 antigen levels are associated with MACE [58].

### 4.4. Neutrophil Extracellular Traps (NETs)

#### 4.4.1. The Association of NETs with Long COVID-19

It was reported that neutrophils undergo a form of cell death distinct from apoptosis or necrosis, termed neutrophil extracellular trap (NET) formation [59]. Neutrophil extracellular traps (NETs), composed of DNA, histones, and antimicrobial proteins such as myeloperoxidase (MPO), neutrophil elastase, cathelicidin, and calprotectin, are released by neutrophils in response to pathogens [60]. NETs are important mediators of tissue damage in inflammatory diseases. The SARS-CoV-2 virus induces the formation of NETs, which is dependent on the virus binding to ACE2 on the neutrophil [61]. Neutrophil elastase, which is found in NETs, can cleave the S protein, resulting in easier SARS-CoV-2 entry into the cell through ACE2, potentially increasing virus infectivity and its ability to stimulate immune responses [62]. Neutrophils and NETs are implicated in thrombosis formation during severe SARS-CoV-2 infection [63]. The production of NETs is increased under the conditions of COVID-19, and their concentration is associated with the severity of the disease and thrombosis [64]. During COVID-19, SARS-CoV-2 triggers complement activation [65]. Subsequently, C3a might activate platelets, while C5a and platelet-derived thrombin induce both neutrophil tissue factor (TF) expression and NETs carrying active TF [65]. These thrombogenic NETs may induce endothelial cell activation leading to TF expression, thus increasing their procoagulant activity. Increases in NET formation during SARS-CoV-2 infection have been linked to ischemic stroke [66].

NET formation levels strongly correlated with illness severity/duration and platelet activation markers, and coagulation factors were significantly reduced upon dexamethasone treatment and recovery [67]. Patients with long COVID maintained higher NET formation levels compared to recovered convalescent patients [67]. The persistence of NETs is associated with pulmonary fibrosis, cardiovascular abnormalities, and neurological dysfunction in long COVID-19 [68].

#### 4.4.2. The Association of NETs with ASCVD

NETs have been found to be present in atherosclerotic lesions [69]. NETs can promote endothelial dysfunction and vascular inflammation, contributing to the initiation and progression of atherosclerotic plaques, by the activation and damage of endothelial cells via type I interferon responses [70] and through the recruitment of other immune cells, mainly macrophages [71]. MPO accumulation in NETs drives further ROS release and the modification of low-density lipoproteins (LDLs) to oxidized LDLs, thus promoting the development of foam cells [72].

### 4.5. Selectins

#### 4.5.1. The Association of Selectins with Long COVID-19

Compared to controls, long COVID-19 patients had increased levels of L-selectin and P-selectin [26]. Another study showed that P-selectin levels were elevated in long COVID-19 patients [73]. L-selectin binds multiple ligands expressed on endothelial cells, while P-selectin interacts exclusively with P-selectin glycoprotein ligand-1 (PSGL-1) on leukocytes [74]. L-selectin binding to the PSGL-1 expressed by leukocytes may mediate neutrophil rolling on stationary leukocytes bound to cytokine-induced endothelial cells [74].

#### 4.5.2. The Association of Selectins with ASCVD

A meta-analysis showed that E-selectin gene polymorphism was associated with an increased risk of ischemic stroke [75]. Another meta-analysis suggested that there is an increase in the risk of CAD conferred by the Ser128Arg polymorphism of the selectin gene (OR, 1.33; 95% CI, 1.04 to 1.69; *p* = 0.02), and the thr715Pro polymorphism may be a factor protecting against myocardial infarction (OR, 0.81; *p* = 0.04) [76]. Selectin gene polymorphisms (A561C, G98T) were also reported to be significantly associated with an increased risk of CAD [77].

## 5. The Association of Long COVID-19 with Atherosclerotic Risk Factors

### 5.1. Diabetes

A systematic review and meta-analysis including 10 articles, comprising 11 retrospective cohorts with a total of 47.1 million participants, showed a 64% greater risk (RR, 1.64, 95% CI, 1.51 to 1.79) of diabetes in patients with COVID-19 compared with non-COVID-19 controls. COVID-19 is strongly associated with the risk of incident diabetes, including both type 1 and type 2 diabetes [78]. It remains undetermined whether the burden of newly detected diabetes during the course of acute COVID-19 persists in the post-acute COVID-19 phase. A pooled analysis of 5,787,027 subjects from four observational studies showed a 59% higher risk of the development of incident diabetes in the post-acute COVID-19 phase versus that in healthy controls (hazard ratio (HR), 1.59; 95% CI, 1.40 to 1.81, *p* < 0.001) [79].

### 5.2. Hypertension

The risk of new-onset hypertension in COVID-19 survivors within one year from the index infection was assessed via a systematic review and meta-analysis of the available data. Overall, 19,293,346 patients were included in this analysis [80]. The pooled analysis revealed that recovered COVID-19 patients presented an increased risk of new-onset hypertension (HR, 1.70; 95% CI, 1.46 to 1.97; *p* < 0.0001) within seven months [80].

### 5.3. Dyslipidemia

In the post-acute phase of SARS-CoV-2 infection, compared with a non-infected contemporary control group, those in the COVID-19 group showed higher risks of incident dyslipidemia, including total cholesterol greater than 200 mg/dL (HR, 1.26; 95% CI, 1.22 to 1.29), triglycerides greater than 150 mg/dL (HR, 1.27; 95% CI, 1.23 to 1.31), LDL cholesterol greater than 130 mg/dL (HR, 1.24; 95% CI, 1.20 to 1.29), and HDL cholesterol lower than 40 mg/dL (HR, 1.20; 95% CI, 1.16 to 1.25) [81]. There was also an increased risk of incident lipid-lowering medication use (HR, 1.54; 95% CI, 1.48 to 1·61) [81]. Post-acute care for those with COVID-19 should involve the paying of attention to dyslipidemia as a potential post-acute sequela of SARS-CoV-2 infection.

## 6. The Association of Long COVID-19 with Chronic Kidney Disease (CKD) and Cardiovascular Diseases

In CKD patients, systemic vascular endothelial damage is observed from an early stage, which could explain the frequent development of CVD in CKD patients [14,82]. Cardiomyocyte is the main factor in cardiac function and the development of heart failure (HF); however, its function is underpinned by non-cardiomyocytes such as vascular endothelial cells [14]. Vascular endothelial cells are important cells for maintaining blood perfusion to myocardial cells. Endothelial dysfunction is a very early event in atherosclerosis.

Therefore, endothelial dysfunction is a crucial determinant in the development and progression of CKD, HF, and ASCVD, such as CAD and ischemic stroke [14]. Considering that vascular endothelial dysfunction is the pathological condition underlying long COVID-19, it is thought that long COVID-19 has a negative impact on CKD, ischemic stroke, and ASCVD.

### 6.1. CKD

Severe COVID-19 is often complicated by acute kidney injury (AKI), which may transition into CKD. SARS-CoV-2-induced endothelial injury initiates platelet activation and platelet–neutrophil interaction, inducing the apoptosis of renal tubular cells and enhancing renal fibrosis, and 15–30% of cases show protracted renal injury, raising the risk of transition from AKI to CKD [83]. The changes in renal function were longitudinally investigated in patients with Omicron COVID-19 for 6 months [84]. Compared with renal function in the hospital, serum creatinine levels at 6 months were increased remarkably; meanwhile, eGFR decreased significantly in all patients [84]. Qi, Z. et al. investigated the clinical and pathological features of each variant of SARS-CoV-2-associated kidney injury [85]. Compared with previous strains of SARS-CoV-2, patients with the Omicron infection showed a favorable prognosis. The tendency to develop CKD is one of the main manifestations of long COVID-19 and deserves attention. The effects of long COVID-19 on kidney function were investigated among patients followed in post-COVID-19 recovery clinics in British Columbia, Canada [86]. There was an estimated 2.96 mL/min/1.73 m^2^ decrease in eGFR within 1 year after COVID-19 contraction, equivalent to a 3.39% reduction from the baseline [86]. More than 40% of patients were at risk of CKD. People with long COVID-19 experienced a substantial decline in eGFR within 1 year from the date of infection. The prevalence of proteinuria appeared to be high. A retrospective, multi-database cohort study of patients with COVID-19 from the Hong Kong Hospital Authority, using the UK Biobank databases, showed that patients with COVID-19 incurred greater risk of end-stage renal disease (HR, 1.76; 95% CI, 1.31 to 2.38) and AKI (HR, 2.14; 95% CI, 1.69 to 2.71) [87].

Using combined data from a GWAS on European ancestry and CKD (n = 117,165) and critical COVID-19 (n = 1,059,456), a bidirectional Mendelian randomization analysis was performed [88]. A significant association of CKD with critical COVID-19 (OR, 1.28; 95% CI, 1.04 to 1.58, *p* = 0.01811) was found. Another study also showed that renal impairment was significant in hospitalized COVID-19 patients. The severity of the disease itself is emphasized as one of the main mechanisms contributing to the occurrence of AKI [89]. The severity of COVID-19 is significantly associated with the development of CKD.

Infection is the second leading cause of death in CKD patients. Adequate humoral and cellular immunity are required to minimize pathogen entry and promote pathogen clearance so as to enable infection control. Vaccination can generate cellular and humoral immunity against specific pathogens and is used to prevent many life-threatening infectious diseases. In the case of SARS-CoV-2 infection, COVID-19 can have considerable detrimental effects on patients with CKD [90]. Humoral and cellular responses to vaccines, including mRNA vaccines, are apparently seen in CKD patients, and the response to vaccination is definitely impaired in CKD patients [91].

### 6.2. HF

From the onset of the pandemic, evidence of cardiac involvement in acute COVID-19 has been abundant. Cardiac presentations ranged from arrhythmias to ischemia, myopericarditis/myocarditis, ventricular dysfunction, acute HF, and cardiogenic shock. SARS-CoV-2 was reportedly detected in endothelial cells and cardiac myocytes. SARS-CoV-2 myocarditis and SARS-CoV-2-infection-induced endothelial injury may lead to the cardiac pathophysiology of long COVID-19 [92]. Hospitalized patients recovering from COVID-19 were characterized by a high prevalence of left ventricular concentric remodeling, predominantly Grade I diastolic dysfunction, and a mild decrease in the longitudinal systolic function. These changes were largely persistent up to the 1-month follow-up [93]. The Hong Kong Hospital Authority and the UK Biobank databases showed that patients with COVID-19 carried a greater risk of HF (HR 1.82; 95% CI 1.65, 2.01) during their post-acute phase of infection [87]. The meta-analysis showed that recovered COVID-19 patients carried an increased risk of incident HF (HR, 1.90; 95% CI, 1.54 to 3.24, *p* < 0.0001) [94]. COVID-19 survivors had an additional 90% risk of developing HF after COVID-19 in the early post-acute phase of COVID-19. The Mendelian randomization study, which investigated the causal association between the genetic predisposition to COVID-19 and long COVID-19, revealed that a genetic predisposition to COVID-19 was significantly causally linked to an increased risk of developing HF [95].

### 6.3. CAD

The Hong Kong Hospital Authority and the UK Biobank databases showed that patients with COVID-19 incurred greater risk of CAD (HR, 1.32; 95% CI, 1.07 to 1.63) and cardiovascular mortality (HR, 2.86; 95% CI, 1.25 to 6.51) during their post-acute phase of infection [87]. Over a mean follow-up of 8.5 months, among recovered COVID-19 patients, acute myocardial infarction occurred in 3.5 cases per 1000 individuals, compared to 2.02 cases per 1000 individuals in the control cohort. COVID-19 patients showed an increased risk of incident acute myocardial infarction (HR, 1.93; 95% CI, 1.65 to 2.26, *p* < 0.0001) [96].

### 6.4. Ischemic Stroke

In a meta-analysis undertaken to assess the risk of ischemic stroke within 1 year after the post-acute phase of COVID-19, over a mean follow-up of 9.2 months, recovered COVID-19 patients presented a higher risk of ischemic stroke (HR, 2.06; 95% CI, 1.75 to 2.41; *p* < 0.0001) compared to people who did not have COVID-19 [97].

## 7. Possible Therapeutic Interventions for Long COVID-19 Considering Endothelial Dysfunction as the Therapeutic Target

Endothelial nitric oxide (NO) synthase (eNOS) produces NO in endothelial cells, and eNOS is closely associated with the regulation of anti-atherogenic processes such as vasorelaxation, an inhibition of the adhesion between leukocytes and endothelial cells, the suppression of the migration and proliferation of vascular smooth muscle cells, and the inhibition of platelet aggregation [98,99,100]. Endothelial dysfunction reduces NO bioavailability and increases oxidative stress [14]. Therefore, NO and antioxidants represent possible therapeutic options for long COVID-19.

In addition to hypolipidemic effects, statins exert pleiotropic effects without a clear relationship with LDL-C levels [101]. The pleiotropic properties of statins, such as an improvement in endothelial function and an increase in the bioavailability of NO [102], as well as anti-inflammatory [103], immunomodulatory, and antioxidant effects [104], may contribute to an improvement in long COVID-19. A recent meta-analysis showed that n-3 polyunsaturated fatty acid (PUFA) supplementation can improve inflammation and endothelial function, as estimated by FMD [105]. Statins and n-3 PUFAs can also offer possible candidates for use in the treatment of long COVID-19.

Beyond lowering plasma glucose levels, sodium-glucose cotransporter 2 inhibitors (SGLT2is) significantly reduce hospitalization for HF and retard the progression of CKD [106,107,108,109,110,111,112]. SGLT2is have been shown to improve endothelial dysfunction, as assessed by FMD, in individuals at a high risk of ASCVD [113,114,115]. The suppression of the development of HF and progression of CKD achieved by SGLT2is might be largely related to their capacity to improve vascular endothelial function [14]. SGLT2is may contribute to treating long COVID-19.

### 7.1. NO

The safety and efficacy of the combined inhalation of NO and molecular hydrogen (H2) in long COVID-19 patients with respiratory manifestations were evaluated [116]. Following the combined inhalation of NO and H2, symptom severity, such as of dyspnea, cough, fatigue, and palpitations, significantly decreased (*p* < 0.005), and other markers of quality of life improved. The distance walked (*p* = 0.01) and the saturation of percutaneous oxygen (*p* = 0.04) in a 6-min walk test, the volumetric blood flow velocity in venules (*p* < 0.001), and oxidative damage (*p* < 0.001) and antioxidant activity (*p* = 0.03) were all improved by the combined inhalation of NO and H2.

### 7.2. Antioxidants

#### 7.2.1. Hydrogen-Rich Water

Hydrogen has potential use as a therapeutic agent in managing long COVID-19 due to its antioxidant and anti-inflammatory properties. In the randomized, single-blind, placebo-controlled study (RCT), hydrogen-rich water was effectively used to alleviate fatigue and improve cardiorespiratory endurance, musculoskeletal function, and sleep quality [117].

#### 7.2.2. L-Arginine with Vitamin C

L-arginine is an amino acid that acts as a substrate for eNOS [118], and it has been previously shown to significantly improve endothelial function in COVID-19 patients [119]. Additionally, the beneficial effects of l-arginine on the regulation of immune responses have been reported [120]. Vitamin C potentiates NO synthesis in cultured human endothelial cells by enhancing eNOS enzymatic activity [121,122,123,124].

After 28 days of l-arginine plus vitamin C supplementation, serum l-arginine concentrations were significantly increased compared with a placebo. This supplement may therefore be proposed as a remedy when used to increase NO bioavailability in people with long COVID-19 [125].

In the LINCOLN (L-Arginine and Vitamin C Improves Long COVID-19) survey, patients were divided into two groups, with a 2:1 ratio: the first group included patients that received l-arginine + vitamin C, whereas the second group received a multivitamin combination (alternative treatment) [126]. The survey included effort perception, measured using the Borg scale. The l-arginine + vitamin C treatment arm showed significantly lower scores compared to patients who had received the multivitamin combination. When examining effort perception, a significantly lower value (*p* < 0.0001) was observed in patients receiving l-arginine + vitamin C compared to that in the alternative treatment arm.

In another RCT, participants were randomized 1:1 to orally receive either a combination of 1.66 g l-arginine plus 500 mg liposomal vitamin C or a placebo twice daily for 28 days [127]. At 28 days, l-arginine plus vitamin C increased the 6-min walk distance (median (interquartile range) +30 (40.5) m; placebo: +0 (75) m, *p* = 0.001) and induced a greater improvement in handgrip strength (+3.4 (7.5) kg) compared with the placebo (+1 (6.6) kg, *p* = 0.03). The FMD was greater in the active group than in the placebo (14.3% (7.3) vs. 9.4% (5.8), *p* = 0.03). At 28 days, fatigue was reported by 2 participants in the active group (8.7%) and by 21 in the placebo group (80.1%; *p* < 0.0001). L-arginine plus vitamin C supplementation improved walking performance, muscle strength, endothelial function, and fatigue in adults with long COVID-19.

#### 7.2.3. Coenzyme Q10 and Alpha Lipoic Acid

The inclusion of mitochondrial nutrients into the diet is important for their clinical antioxidant effects. An association of the supplementation of coenzyme Q10 and alpha lipoic acid with long COVID-19 symptoms was examined [128]. A Fatigue Severity Scale (FSS) complete response was reached in 53.5% of patients in the treatment group and in 3.5% of patients in the control group. A reduction in FSS score < 20% from baseline (non-response) was observed in 9.5% of patients in the treatment group and in 25.9% of patients in the control group (*p* < 0.0001).

### 7.3. Statins

A prospective cohort study was performed to explore the effects of statins on long-term respiratory symptoms and pulmonary fibrosis in COVID-19 patients with diabetes [129]. Non-statin patients with >5 years of diabetes were more likely to exhibit a significantly higher pulmonary fibrosis score during the follow-up period as compared to the statin group. The use of statins was associated with a lower risk of developing chronic cough and dyspnea in diabetic patients with COVID-19 and may have reduced pulmonary fibrosis associated with COVID-19 in patients with long-term (>5 years) diabetes. Another study showed that a 6-week treatment with statins plus angiotensin II type 1 receptor blockers improved clinical symptoms in patients with long COVID-19 [130].

### 7.4. N-3 PUFAs

Two relatively large RCTs are currently underway, testing the hypothesis that the treatment of severe forms of COVID-19 with n-3 PUFAs is beneficial [131]. However, clinical interventional studies investigating the role of n-3 PUFAs in individuals with long COVID-19 are currently not available. Nonetheless, due to the general favorableness of their profiles when viewed from various standpoints (psychiatric and cardiovascular benefits), n-3 PUFAs can be considered a potential health supplement that help maintain physical and mental health in patients with long COVID-19.

### 7.5. SGLT2is

In patients with COVID-19, proinflammatory cytokines induced a redox-sensitive upregulation of SGLT2 expression in endothelial cells, which in turn promoted endothelial injury, senescence, platelet adhesion, aggregation, and thrombin generation [132]. SGLT2 inhibition emerged as an attractive strategy to restore vascular homeostasis in COVID-19.

Furthermore, SGLT2is suppress the progression of CKD and the development of HF and ASCVD, which is beneficial to preventing future pandemics of such symptoms induced by long COVID-19.

## 8. Limitations of This Review

The clinical symptoms related to long COVID-19 are incredibly heterogeneous and include the respiratory and gastrointestinal tracts, joints, the central and peripheral nervous system, bone marrow, the endocrine system, etc. [133]. Respiratory tract symptoms such as dyspnea, cough, and reduced lung capacity, neurological symptoms such as concentration disorder, anosmia, and dysgeusia, and other symptoms such as joint pain, chronic fatigue, and chest pain are commonly observed in patients with long COVID-19 [134].

Since long COVID-19 involves a broad spectrum of non-specific manifestations, it may not be possible to explain the pathology of long COVID-19 by recourse to only endothelial dysfunction. Furthermore, the therapeutic interventions described above may not necessarily improve the pathology of long COVID-19.

## 9. Conclusions

**Figure 3:**
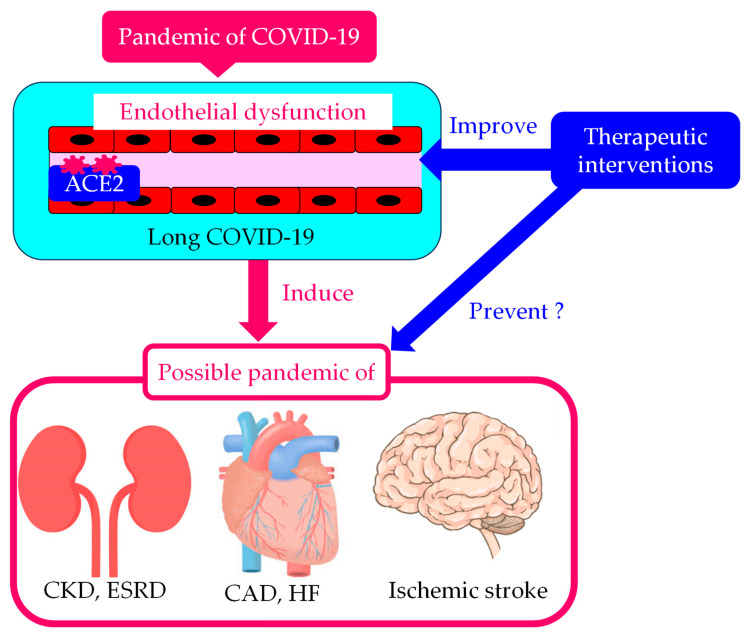
Possible induction of widespread CKD, cardiovascular disease, and ischemic stroke due to endothelial dysfunction in long COVID-19, and the prevention of such problems using therapeutic interventions for long COVID-19 considering endothelial dysfunction as the therapeutic target. ACE2, angiotensin-converting enzyme 2; CAD, coronary artery disease; CKD, chronic kidney disease; ESRD, end-stage renal disease; HF, heart failure.

The COVID-19 pandemic created a great number of long COVID-19 patients. Endothelial dysfunction in long COVID-19 can induce widespread problems of CKD, CAD, HF, and ischemic stroke (Figure 3). Therapeutic interventions for long COVID-19 taking endothelial dysfunction as the therapeutic target are required to prevent pandemics of such diseases, these being the worst-case scenario.
